# 

**Published:** 1881-08

**Authors:** 


A MONTHLY JOURNAL,

DEVOTED TO

### Medical, Surgical, Obstetrical, Dental, Hygienical and Popular Sciences.

EDITED BY
HARVEY L. BYRD, A. M., M. D.,
AND
BASIL M. WILKERSON, D. D. S., M. D.

"Altera poscit opem res, et conjurat amice."

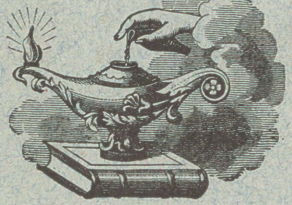

NEW YORK:
*Published by B. M. WILKERSON, Proprietor,*
Office, 20 Courtlandt Street.
Wm. Miller, Business Manager.

$3.00 Per Annum in Advance. - - Single Copies 30 Cents.

ENTERED AT THE POST OFFICE, NEW YORK CITY, AS SECOND-CLASS MATTER.

